# Uniform Semiclassical Instanton Rate Theory

**DOI:** 10.1021/acs.jpclett.3c02779

**Published:** 2023-10-31

**Authors:** Sameernandan Upadhyayula, Eli Pollak

**Affiliations:** Chemical and Biological Physics Department Weizmann Institute of Science, Rehovoth 76100, Israel

## Abstract

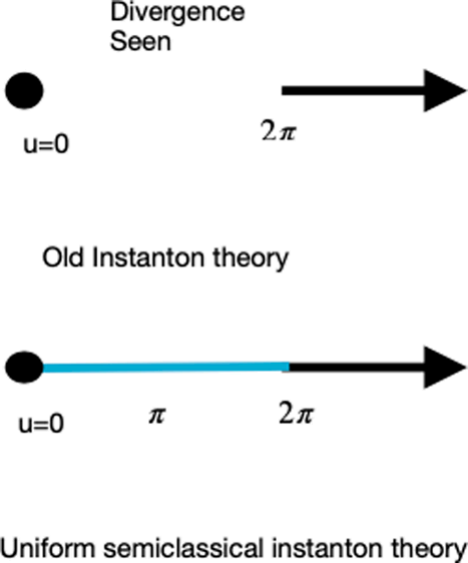

The instanton expression for the thermal transmission probability through a one-dimensional barrier is derived by using the uniform semiclassical energy-dependent transmission coefficient of Kemble. The resulting theory does not diverge at the “crossover temperature” but changes smoothly. The temperature-dependent energy of the instanton is the same as the barrier height when ℏ*βω*^‡^ = π and not 2π as in the “standard” instanton theory. The concept of a crossover temperature between tunneling and thermal activation, based on the divergence of the instanton rate, is obsolete. The theory is improved by assuring that at high energy when the energy-dependent transmission coefficient approaches unity the integrand decays exponentially as dictated by the Boltzmann factor and not as a Gaussian. This ensures that at sufficiently high temperatures the uniform theory reduces to the classical. Application to Eckart barriers demonstrates that the uniform theory provides a good estimate of the numerically exact result over the whole temperature range.

Over 50 years have passed since Miller discovered what is nowadays known as the instanton—a periodic orbit on the inverted potential energy surface at temperature *T*, with period ℏβ (β = 1/*k*_*B*_*T*).^1,2^ Four years later Miller derived the instanton expression for thermal rates.^3^ He assumed that the energy-dependent transmission probability *T*_sc_(*E*) through a barrier at energies below the barrier height *V*^‡^ is as derived via semiclassics, that is
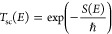
1*S*(*E*) is the barrier penetration integral, which we will henceforth, for the sake of brevity, refer to as the “action” of the instanton trajectory at energy *E*. Considering for simplicity one-dimensional systems, the quantum thermal transmission factor (relative to the classical) also known as the tunneling correction factor is defined as
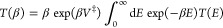
2with *T*(*E*) being the exact quantum transmission factor. After inserting the semiclassical approximation eq 1 into eq 2, Miller estimated the integral using the steepest descents method. The steepest descent condition is
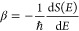
3and it identifies the instanton as the classical trajectory with energy *E*_β_ whose period is ℏβ. The resulting expression for the transmission factor is
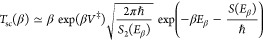
4with *S*_2_(*E*_β_) being the second derivative of the instanton action with respect to the energy, at the steepest descent energy *E*_β_.

This theory, with all its beauty and simplicity, has some problems that have eluded solution. Foremost, if the potential around the barrier top has a parabolic shape with barrier frequency ω^‡^, then the instanton action will vanish when *E*_β_ = *V*^‡^ and this occurs precisely when ℏ*βω*^‡^ = 2π. This leads to a divergence since the second derivative of the action vanishes at this point. Second, for higher temperatures, there is no longer a solution for the steepest descent equation apart from the trivial one, which is the barrier top with zero action. In practice, one replaces the instanton approximation with a parabolic barrier estimate, which also diverges at the “crossover” temperature at which ℏ*βω*^‡^ = 2π. This temperature is termed “crossover” since it distinguishes between the low-temperature region where transmission occurs via tunneling and there exists an instanton with finite action and the high-temperature region where the transmission is identified with above-barrier classical-like motion. All this implies that the instanton solution has an unnatural discontinuity at the crossover temperature.^4^

Miller’s instanton was rediscovered by Coleman and Callan^5,6^ using a very different methodology known as the imaginary free energy (ImF) method, invented originally by Langer^7^ to estimate on some model systems the condensation point associated with a first-order phase transition and later used to estimate reaction rates.^8^ Affleck noted^4^ that the ImF method when applied to thermal rates has a different prefactor below and above the crossover temperature and proceeded to show how the divergence at the crossover temperature may be bridged by expanding the action to second order in the energy when it is close to the barrier energy. Many other efforts have been made over the years to overcome the problem of discontinuity at the crossover temperature.^10−12^ The early history of “instanton theory” is excellently described in the review article of Hänggi et al.^9^

In particular, within the chemistry community, instanton-based rate theory has seen a large revival of interest, since it demands the computation of only a single periodic orbit and its vicinity in phase space and so is amenable to on-the-fly computations.^13−15^ It is not trivial to locate the instanton orbit in multidimensional systems since it is rather unstable, however numerical formulas have been devised^13,16^ and the method is now a standard tool used for studying tunneling in large complex systems. In recent years, Richardson and co-workers have generalized the theory so that it is amenable for the study of nonadiabatic tunneling systems.^17−20^

In view of this, it is of interest to provide a formulation of the instanton rate theory that does not suffer from discontinuities, is accurate, and is not more difficult to apply in practice than the ”standard” instanton theory. This is the purpose of this Letter. Our formulation starts with the uniform JWKB semiclassical formula for the energy-dependent transmission coefficient
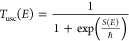
5originated by Kemble^21^ and later formalized by Nanny and Per Olof Fröman.^22^ Using this uniform semiclassical energy-dependent transmission coefficient we rewrite the thermal transmission coefficient as
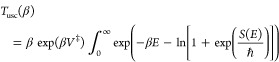
6and proceed to estimate this expression using steepest descents. For this purpose, we define a “uniform” thermal action function
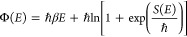
7

The steepest descent condition is readily seen to be
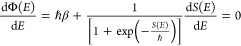
8and this defines the thermal energy *E*_β_ of the instanton. In the deep tunneling regime, where *S*(*E*_β_) ≫ 1, this condition reduces to the standard one (eq 3). However, especially at temperatures that are higher than the standard crossover temperature ℏ*βω*^‡^ = 2π the steepest descent thermal energy *E*_β_ differs significantly from that obtained from the “standard” steepest descent condition as written in eq 3. In the infinite temperature limit (ℏβ → 0) the action *S*(*E*) → – ∞ so that *E*_β_ → ∞. The steepest descent estimate for the uniform temperature-dependent transmission probability is readily found to be
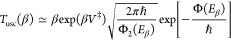
9where Φ_2_ denotes the second derivative of the uniform action at the steepest descent point with respect to the energy and one notes that it has two contributions
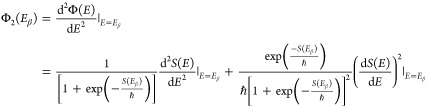
10Even if the second derivative of the action of the instanton trajectory vanishes, which is the case for the parabolic barrier, the second derivative of the uniform action does not, indicating that this uniform theory does not lead to divergences.

To obtain a feeling for the differences between the uniform expression as compared to the “standard” instanton theory, it is instructive to consider the high-temperature limit in which the action around the barrier energy^4^ is well approximated by
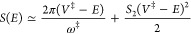
11. Using the uniform semiclassical theory, the steepest descent eq 8 becomes
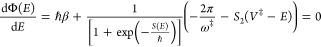
12Since *S*(*V*^‡^) = 0, the solution *E*_β_ = *V*^‡^ occurs at the reduced temperature β_*c*_
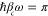
13The so-called crossover temperature is thus a factor of 2 higher! There is no divergence at this higher temperature, and the uniform thermal transmission coefficient obtained from eq 9 is
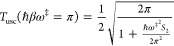
14

The parabolic barrier estimate of the transmission coefficient is well-known
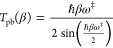
15Comparing the uniform semiclassical estimate to the parabolic barrier estimate at β_*c*_ one finds
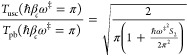
16For a purely parabolic barrier *S*_2_ = 0 the uniform instanton result for the rate is only somewhat lower than the exact result ().

A central difference between the uniform steepest descent energy and the “standard” energy is due to the term [1+ exp(−(*S*(*E*)/ℏ))]^−1^ which causes a reduction of the energy *E*_β_ found in the uniform theory as compared to the “standard” one. This is shown in Figure 1 where we plot the temperature-dependent steepest descent energy as a function of ℏ*βω*^‡^ for the “standard” and uniform instanton theories using the Eckart barrier as a model. For the standard instanton theory, the value of *E*_β_ rises steeply above the crossover temperature ℏ*βω*^‡^ = 2π. However, in the uniform semiclassical instanton theory, this rise is gradual, and hence the instanton trajectory will contribute to the rate at much higher temperatures than the “standard” instanton.

**Figure 1:**
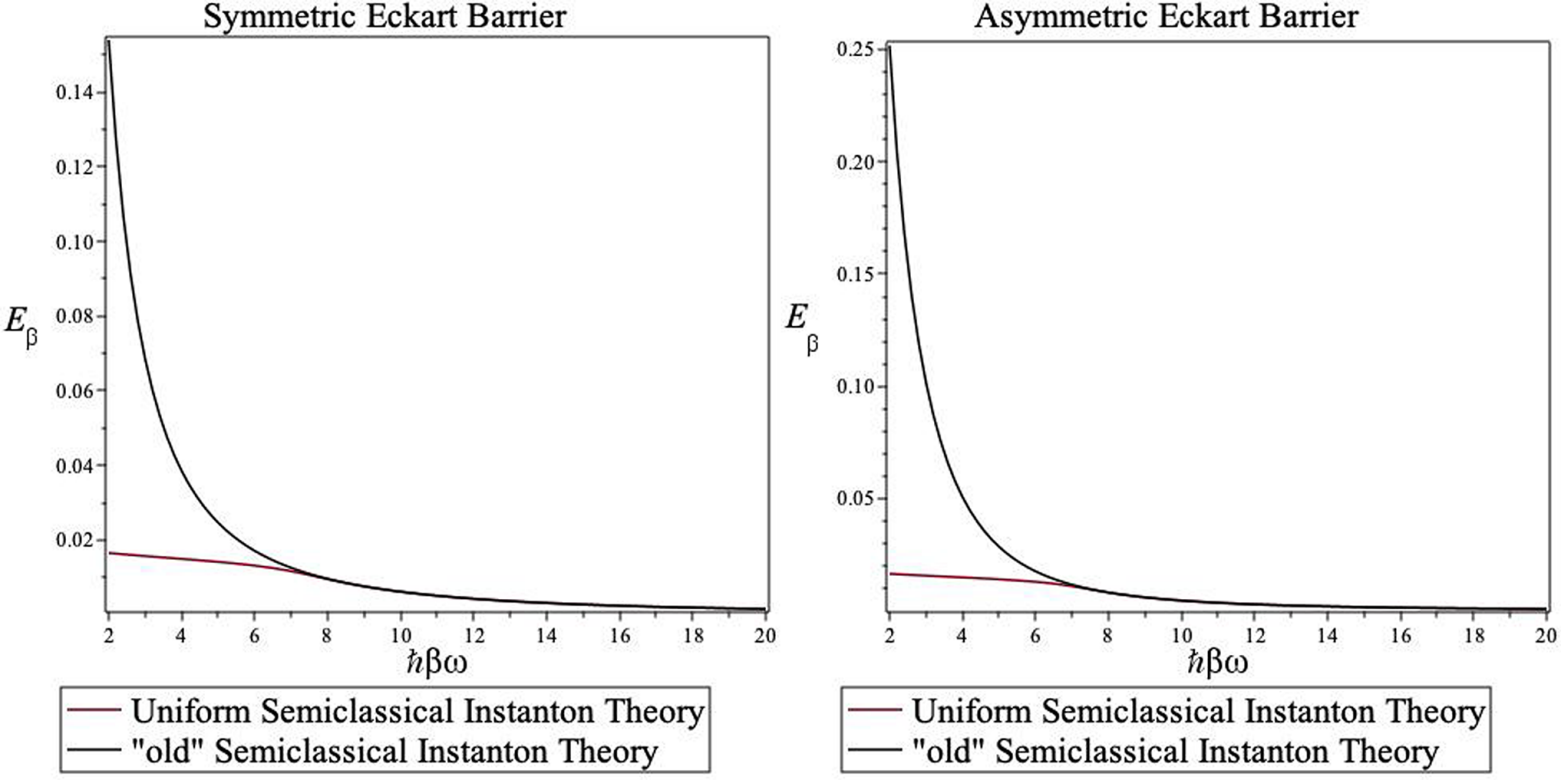
Dependence of the steepest descent energy *E*_β_ on the inverse (reduced) temperature ℏ*βω* for the symmetric (left panel) and asymmetric (right panel) Eckart barriers. For further details, see the text.

There is, however, a significant drawback to the steepest descent estimate. For scattering through a barrier, we know that at sufficiently high energy the energy-dependent transmission coefficient goes to unity. This implies that at high energy the uniform action Φ(*E*) will be linear in the energy. The steepest descent approximation is based on representing the exponent in the temperature-dependent transmission probability as quadratic about the point of steepest descent
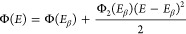
17and not linear. This qualitatively incorrect energy dependence will thus lead to a steepest descent estimate that is too low, since the Gaussian used in the steepest descent estimate decays too quickly. This is shown in Figure 2 for a symmetric Eckart barrier model and in Figure 3 for an asymmetric one. Here we plot the integrands involved in the thermal average (exp(−*βE*)*T*_usc_(*E*)). The solid line is the exact semiclassical integrand, and the dashed line is its Gaussian steepest descent approximation. One notes that especially in the high-temperature case, the long linear exponential tail is higher than its Gaussian steepest descent approximation, irrespective of the shape of the barrier.

**Figure 2:**
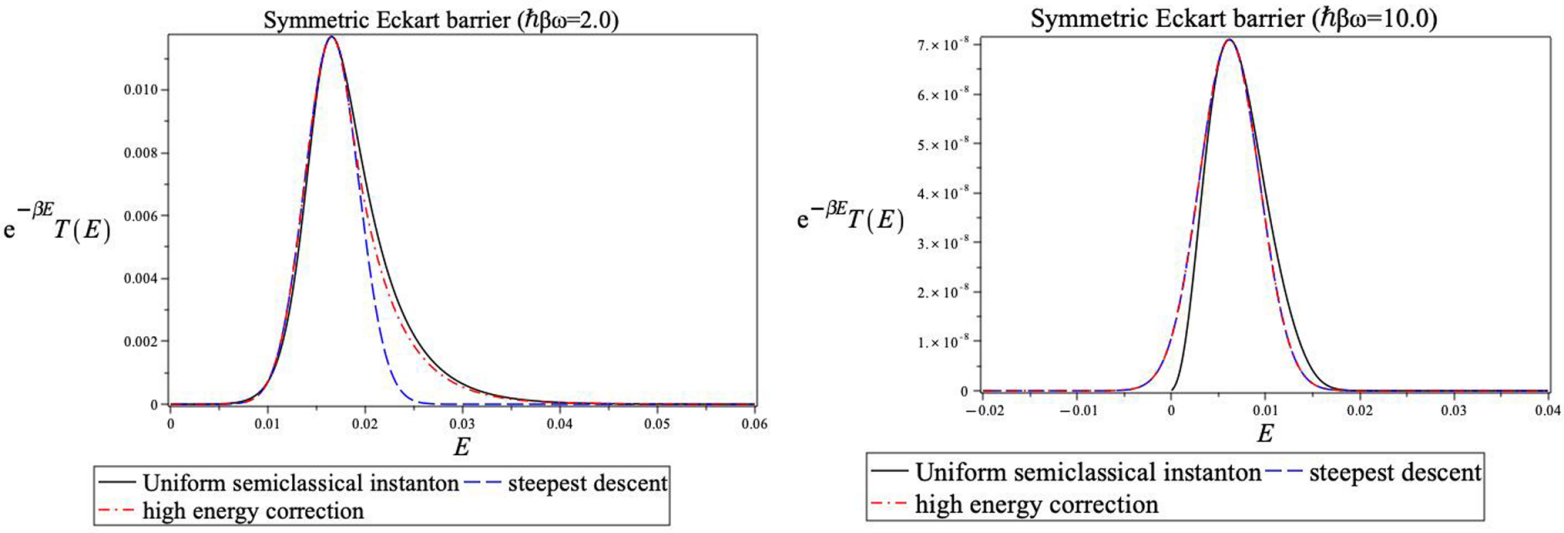
Comparison of thermally weighted integrands with the exact uniform semiclassical integrand (solid line) for a symmetric Eckart barrier. The dashed line uses the Gaussian integrand (eq 17) in the exponent of the transmission probability, while the dashed-dotted line uses the high energy corrected Gaussian integrand (eq 19) in the exponent.

**Figure 3:**
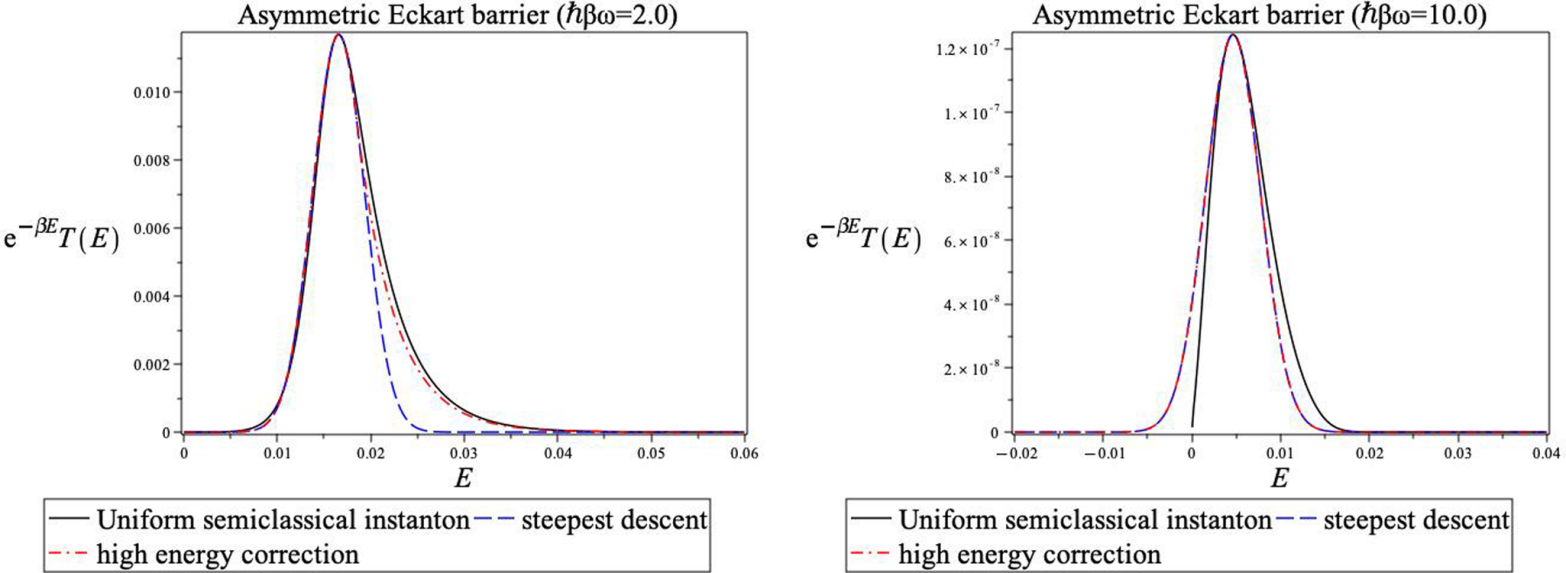
Comparison of integrands of various expressions involved for the asymmetric Eckart barrier. The notation is as shown in Figure 2.

It is straightforward to correct for this, by matching the exponential decay to the quadratic decay such that for energies greater than some energy *E*_β_^*^, the reduced action takes the form
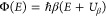
18The two parameters *E*_β_^*^ and *U*_β_ are determined by demanding continuity of the function Φ(*E*) and its first derivative with respect to the energy. Continuity of the function implies
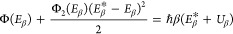
19and continuity of the first derivative leads to the condition
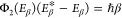
20We thus find that
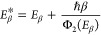
21and
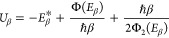
22Using this modified form of the integrand one finds that the modified steepest descent approximation becomes
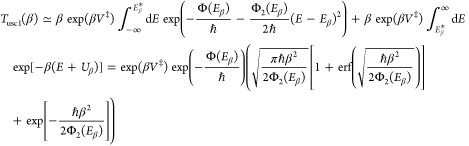
23This high-energy modified uniform expression is the final central formal result of this Letter. Evaluating it does not increase the computational expense since all that is needed is the second derivative of the action and this is already included in the ”pure” steepest descent estimate.

To get a feeling for how important the high energy modification is, we consider what happens at the revised crossover temperature ℏβ_*c*_ω^‡^ = π. Setting *S*_2_ = 0 one finds
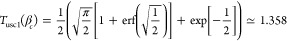
24With the high energy correction, the uniform instanton result is now off by a factor ∼0.86 compared to the parabolic barrier result, a substantial improvement as compared to the pure steepest descent value of ∼0.80. Not less important is that now, in the limit that β → 0 the uniform theory reduces to the classical, and the transmission factor goes to unity.

It is instructive to consider the application of the uniform instanton theory to the symmetric and asymmetric Eckart barrier. The Hamiltonian of the symmetric Eckart barrier is
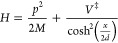
25The barrier frequency is . The exact energy-dependent transmission probability is
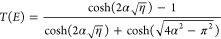
26where  measures the width of the barrier and  is the reduced energy. The energy-dependent action is
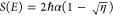
27The parameter that defines the dynamics is chosen such that *V*^‡^/(ℏω^‡^) = 6/π. The results for the one-dimensional thermal transmission coefficient are presented in Table 1 and Figure 4. Columns 2–6 correspond to the exact quantum thermal transmission factor (*T*_exact_), the numerically exact uniform semiclassical transmission factor obtained by numerical integration of the energy-dependent uniform semiclassical transmission factor of eq 5 (*T*_usc,num_), the uniform instanton result with high energy modification, obtained through eq 23 (*T*_usc1_), the uniform semiclassical instanton result obtained from steepest descent as given in eq 9 (*T*_usc_), and the “standard” instanton result of eq 4 (*T*_sc_), respectively.

**Figure 4:**
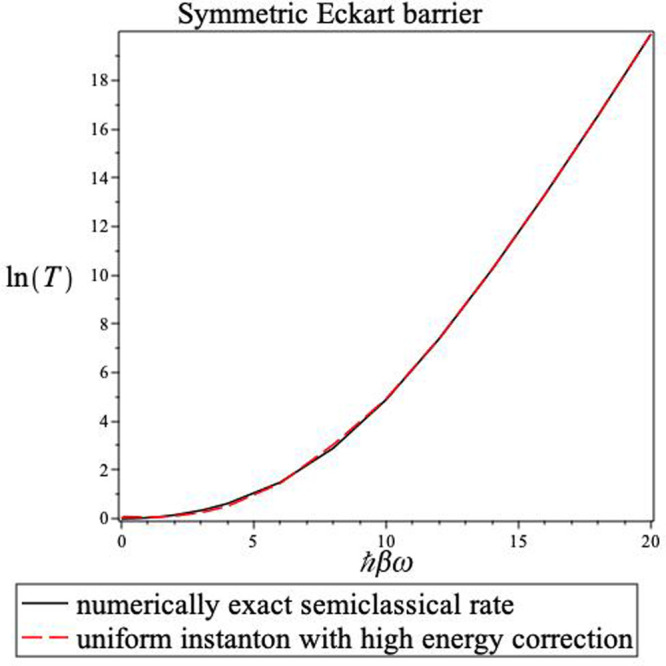
Inverse temperature dependence of the transmission probability for a symmetric Eckart barrier. The solid line is the result obtained from the numerical integration of eq 2 using the uniform semiclassical energy-dependent transmission probability. The dashed line shows the steepest descent instanton result with the high energy correction, using eq 23. Note the accuracy of the steepest descent estimate at all temperatures and the fact that there is no divergence when ℏ*βω*^‡^ = 2π.

**Table 1:** Transmission Coefficients for a Symmetric Eckart Barrier

ℏ*βω*^‡^	*T*_exact_	*T*_usc,num_	*T*_usc1_	*T*_usc,instanton_	*T*_sc,instanton_
0.5	1.016	1.004	1.069	0.570	–
1.0	1.064	1.030	1.055	0.708	–
1.5	1.129	1.077	1.065	0.808	–
2	1.224	1.149	1.104	0.906	–
3	1.525	1.391	1.278	1.157	–
π	1.583	1.439	1.316	1.204	–
4	2.071	1.839	1.651	1.581	–
6	5.198	4.414	4.257	4.250	–
8	21.769	17.973	20.766	20.766	21.995
10	161.905	132.174	136.013	136.013	136.186
12	1973.338	1606.829	1613.027	1613.004	1613.149
14	34057.358	27735.744	27750.669	27749.516	27749.885
16	7.404 × 10^5^	6.036 × 10^5^	6.037 × 10^5^	6.036 × 10^5^	6.036 × 10^5^
18	1.882 × 10^7^	1.537 × 10^7^	1.537 × 10^7^	1.537 × 10^7^	1.537 × 10^7^
20	5.343 × 10^8^	4.374 × 10^8^	4.374 × 10^8^	4.373 × 10^8^	4.373 × 10^8^

Any semiclassical steepest descent approximation cannot give an answer that is more accurate than the numerically evaluated semiclassical result. Although *T*_usc,num_ is quite close to the exact quantum result, it is not perfect. However, we find that the uniform semiclassical instanton with the high energy correction (*T*_usc1_) is within ten percent of the numerical semiclassical result over the whole temperature range as also shown in Figure 4. The present theory gives a smooth result for all temperatures; there are no divergences that must be considered. Inspection of Figure 1 shows that for the “standard” instanton theory, the value of *E*_β_ rises steeply above the crossover temperature while in the uniform semiclassical instanton theory, this rise is gradual and hence the results are comparable to the exact semiclassical results even in the high-temperature limit.

The significant improvement of the uniform instanton theory results at high temperatures coming from the high energy correction can be understood from Figure 2. One notes that the dashed-dotted line, which shows the combination of the Gaussian and linear high energy estimate, especially at high temperatures, compensates for the Gaussian tail which decays too rapidly. Including the high energy correction significantly improves the estimate. The same occurs also at lower temperatures; however, due to the low transmission probability, the linear exponential kicks in only at rather high energy so that its contribution to the overall rate becomes small.

The Hamiltonian for the asymmetric Eckart barrier is somewhat more involved
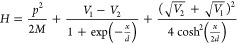
28The barrier is located at , the barrier height is *V*^‡^ = *V*_1_ and the exoergicity is defined as *ΔV* = *V*_2_ – *V*_1_. The transmission coefficient is completely determined in terms of two parameters. One is the ratio of the barrier height to the barrier frequency, chosen such that *V*^‡^/(ℏω^‡^) = 6/π, and the other is the asymmetry ratio *V*_2_/*V*_1_ = 4. For this case, the barrier frequency is related to the barrier height by .

The exact quantum energy-dependent transmission probability for the asymmetric case is
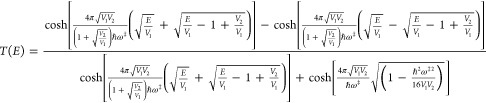
29

The classical action of the instanton is
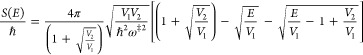
30The results for the 1-D transmission coefficients are presented in Table 2 (where the notation is as in Table 1) and plotted in Figure 5.

**Figure 5:**
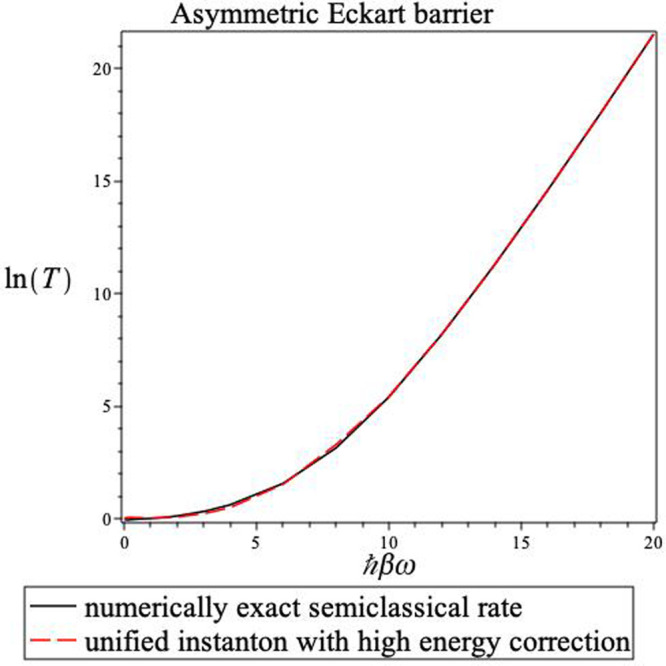
Inverse temperature dependence of the transmission probability for an asymmetric Eckart barrier. The solid line is the result obtained from the numerical integration of eq 2 using the uniform semiclassical energy-dependent transmission probability. The dashed line shows the steepest descent instanton result with the high-energy correction using eq 23. Note the accuracy of the steepest descent estimate at all temperatures and the fact that there is no divergence when ℏ*βω*^‡^ = 2π.

**Table 2:** Transmission Coefficients for an Asymmetric Eckart Barrier

ℏ*βω*^‡^	*T*_exact_	*T*_usc,num_	*T*_usc1_	*T*_usc,instanton_	*T*_sc,instanton_
0.5	1.014	1.006	1.068	0.552	–
1.0	1.049	1.033	1.056	0.707	–
1.5	1.109	1.082	1.067	0.808	–
2	1.195	1.157	1.106	0.909	–
3	1.480	1.412	1.287	1.167	–
π	1.536	1.464	1.326	1.215	–
4	2.014	1.896	1.679	1.612	–
6	5.322	4.895	4.725	4.721	–
8	26.097	23.680	27.459	27.459	28.063
10	251.558	227.231	232.317	232.316	232.395
12	4067.815	3672.367	3689.416	3689.242	3689.348
14	90557.643	81795.631	81934.955	81918.252	81918.719
16	2.445 × 10^6^	2.210 × 10^6^	2.211 × 10^6^	2.210 × 10^6^	2.210 × 10^6^
18	7.466 × 10^7^	6.759 × 10^7^	6.754 × 10^7^	6.746 × 10^7^	6.746 × 10^7^
20	2.476 × 10^9^	2.245 × 10^9^	2.242 × 10^9^	2.237 × 10^9^	2.237 × 10^9^

The results found for the asymmetric Eckart barrier are qualitatively similar to those found in the symmetric case. Here too, the numerical semiclassical results are a good approximation of the exact transmission coefficients. The test of the instanton approximations is then to compare them with the numerical semiclassical results rather than the exact results. Again, we see that the high energy corrected approximation is rather accurate for the whole temperature range. There are no divergences and there are no special difficulties in the high-temperature region.

The uniform high energy modified semiclassical instanton theory developed in this article leads to an interesting change in our understanding of instantons and thermal transmission coefficients. The so-called crossover temperature is seen to be a misnomer; there are no divergences in the uniform instanton theory, and consequently, there is no clearly defined crossover temperature. This resolves a long-standing difficulty when considering the parabolic barrier. We know that tunneling is a major feature of the parabolic barrier transmission coefficient and yet the crossover temperature defined by ℏ*βω*^‡^ = 2π would seem to indicate that at all temperatures, the parabolic barrier dynamics is above barrier crossing. In the present uniform theory, this is no longer so. Even for the parabolic barrier, there exists a nontrivial instanton trajectory and it determines the transmission coefficient when using the steepest descent estimate quite accurately.

This does not mean that the concept of crossover temperatures is useless. For example, one could define the crossover temperature as the temperature at which the instanton energy is the same as the barrier height. In the “old” theory this occurs at ℏ*βω*^‡^ = 2π but in the uniform theory presented here, it occurs when ℏ*βω*^‡^ = π. This makes physical sense; however, it too is at best an approximation, since we know that even the numerical semiclassical theory is not exact.

Can one do better with the uniform instanton method presented here? Emphatically, yes. The numerical instanton theory, based on the classical action, is known in the literature as VPT0 (Vibrational Perturbation Theory 0). It is not exact in the high-temperature limit. As shown in the literature,^23^ the VPT2 theory^24,25^ does give the correct leading order correction in ℏ^2^ due to the addition of ℏ^2^ dependent terms to the classical action. The conclusion is that perhaps a more exact semiclassical theory would be to employ the VPT2 action rather than the classical action. One could repeat the steepest descent analysis of the present Letter and obtain the “VPT2 instanton theory”, which will most likely give an improved estimate for the exact transmission coefficient, especially at high temperatures. Continuing in this vein, a better approximation for the action of the Eckart barrier is the Yasumori approximation,^26^ which would then lead to an even more accurate instanton theory.

This Letter has been limited to one dimension, but this is not a serious limitation. Generalization of the present uniform instanton theory to many dimensions would follow the same lines as those in the “standard” instanton theory. The only difference would be in the treatment of motion along the periodic orbit. The steepest descent energy would change, but otherwise, the formal multidimensional result would be the same.
